# Respiratory failure as the prominent manifestation of entecavir-associated mitochondrial myopathy: a case report

**DOI:** 10.1186/s12879-022-07159-y

**Published:** 2022-02-24

**Authors:** Xiao Lin, Aixin Song, Sujun Zheng, Xinyue Chen

**Affiliations:** First Department of Liver Disease Center, Beijing Youan Hospital, Capital Medical University, Beijing, 100069 China

**Keywords:** Entecavir, Respiratory failure, Myasthenia, Dysosmia, Mitochondrial myopathy

## Abstract

**Background:**

Mitochondrial myopathy caused by the long-term use of nucleos(t)ide analogue in patients with chronic hepatitis B (CHB) is mostly characterized by myasthenia and myalgia. Cases with respiratory failure as the prominent manifestation and multisystem symptoms have not been reported.

**Case report:**

We report a case of mitochondrial myopathy associated with the long-term use of entecavir for CHB. The patient was a 54-year-old male who was treated with entecavir for 9 years. During the treatment, hepatitis B virus (HBV) DNA was negative and liver function was normal. However, generalized fatigue, poor appetite, dysosmia and other discomforts gradually presented starting at the 5^th^ year of treatment, and respiratory failure was the prominent manifestation in the later stage of disease progression. The diagnosis was based on histopathology examination. The dysosmia, hypoxemia and digestive tract symptoms were gradually improved after withdrawal of entecavir.

**Discussion:**

Mitochondrial myopathy is a rare side effect of entecavir and can be diagnosed by muscle biopsy. Although the incidence is extremely low, but the severe cases can lead to respiratory failure. We should receive adequate attention in clinical practice.

## Background

At present, the efficacy and safety of the long-term use of nucleos(t)ide analogue (NAs) in patients with chronic hepatitis B (CHB) are widely recognized, but there are also rare adverse reactions, such as NAs-related mitochondrial myopathy with symptoms such as myasthenia and myalgia [1–4]. Recently, we observed a case of NAs-related mitochondrial myopathy that had respiratory failure as the prominent manifestation and was accompanied by fatigue, poor appetite, dysosmia and other multisystem symptoms after the use of entecavir for 5 years. Additionally, we performed a literature review of other cases of entecavir-related myopathy in patients with CHB.

## Case presentation

The patient was a 54-year-old male with a history of CHB for more than 20 years. In April 2011, due to abnormal liver function [alanine aminotransferase (ALT) 80–160 U/L], hepatitis B virus (HBV) DNA 10E + 07 copies/ml, hepatitis B surface antigen (HBsAg) and hepatitis B e antigen (HBeAg) positive, he was diagnosed with HBeAg-positive CHB and started using entecavir (0.5 mg/d). Retests performed after 24 weeks of treatment revealed HBV DNA < 500 copies/ml and normal ALT. The patient continued to take entecavir and attended regular follow-up visits. Starting in May 2016, the patient gradually presented fatigue and poor appetite, and he visited the local hospital multiple times for medical care. There were no positive signs upon physical examination. Ancillary examination results were as follows: blood routine, liver and kidney functions and electrocardiogram were normal, and HBV DNA was less than 100 copies/ml. No special treatment was administered. In December 2018, the patient experienced the onset of dysosmia. In March 2019, the patient experienced a recurrent cough, expectoration with white sputum and with mild difficulty breathing after upper respiratory tract infection. No fever or other complaints were observed. But the symptoms were mostly tolerable and required no further treatment.

In August 2019, he had completely lost his sense of smell and visited the Department of Otolaryngology, Beijing Tongren Hospital, for treatment. The diagnostic work-up comprised nasal endoscopy, computed tomography (CT) and magnetic resonance imaging (MRI) of the sinuses. The results indicated that the nasal septum was bent to the right, and there was no nasal mucosal edema, nasal polyps or space-occupying lesions. In terms of the dysosmia, no special treatment was given. The patient’s fatigue and poor appetite were aggravated during the same period, and his weight had decreased by 3 kg in the past year (his weight continued to decrease by approximately 5 kg until July 2020). Gastroscopy and colonoscopy were performed at the local hospital. These examinations revealed only colonic polyps, which were clamped off with forceps under an endoscope. No other abnormalities were found. In December 2019, the patient was hospitalized at the local hospital due to aggravated cough and expectoration with white sputum accompanied by shortness of breath. The laboratory test results were as follows: arterial blood gas analysis: pH 7.38 (7.35–7.45), arterial oxygen partial pressure (PaO_2_) 61.8 mmHg↓ (80–100), arterial carbon dioxide partial pressure (PaCO_2_) 65.7 mmHg↑ (32–45). Serum muscle zymogram: creatine kinase-myocardial band (CK-MB) 35.9 U/L ↑ (0–25), lactate dehydrogenase (LDH) 253 U/L↑ (120–250), hydroxybutyrate dehydrogenase (HBDH) 237 U/L ↑ (72–182), creatine kinase (CK) 123.9 U/L (50–310). Lung function: forced expiratory volume in 1 s (FEV1)/forced vital capacity (FVC) 67% (65.9–89.5), FEV1 1.60 L (51% of the predicted value), FVC 2.38 L (61% of the predicted value), and a negative bronchial dilation test, indicated a possible restrictive pulmonary ventilation disorder. Chest CT showed mild consolidation in the right lower lung. Blood routine, C-reactive protein, liver and kidney functions and echocardiography were normal, and HBV DNA was less than 100 IU/ml. The patient was diagnosed with chronic obstructive pulmonary disease and type II respiratory failure. Theophylline and salbutamol were given, but their efficacy was poor. Piperacillin sulbactam combined with levofloxacin were added to anti-infective treatment, the cough and expectoration were improved for a while. But these symptoms continued to recur after treatment withdrawal, and the patient’s shortness of breath was not obviously improved.

On January 14, 2020, the patient visited the Department of Respiratory Medicine, Peking Union Medical College Hospital, and underwent arterial blood gas analysis. The results were as follows: resting state: pH 7.35, PaO_2_ 66 mmHg↓, PaCO_2_ 65 mmHg↑, oxygen saturation (SO_2_) 92.1%↓, lactate 4.3 mmol/L ↑ (0.5–1.6). After deep breathing: pH 7.40, PaO_2_ 107 mmHg, PaCO_2_ 50 mmHg ↑, SO_2_ 98.3%, lactate 6.5 mmol/L ↑. After the 6-min walking test (walking 510 m): pH 7.29↓, PaO_2_ 74 mmHg ↓, PaCO_2_ 56 mmHg ↑, SO_2_ 93.7% ↓, lactate 11.5 mmol/L ↑. The patient’s clinical symptoms could not be explained by the blood gas test results and the previous lung imaging findings. The blood lactate increased significantly after exercise. Considering that mitochondria closely related to lactate metabolism might have undergone pathological changes, the diagnosis of neuromuscular myopathy could not be ruled out. Additional related examinations were carried out as follows: blood routine, urinalysis and stool tests showed basically normal results; the liver function, coagulation function, immunoglobulin (IgG/IgM/IgA), complements (C3/C4), anti-nuclear antibody, extractable nuclear antigen, anti-neutrophil cytoplasmic antibodies spectra, erythrocyte sedimentation rate and C-reactive protein were all within normal ranges; HBV DNA < 100 IU/ml. The Serum muscle zymogram showed CK-MB 20.1 μg/L ↑ (0–3.6), LDH 372 U/L ↑, CK 165 U/L (24–195). No pathological changes were detected with electrocardiography and echocardiography. The lung function test was repeated, and the results indicated FEV1/FVC 85.85%, FEV1 1.82 L (58% of the predicted value), FVC 2.12 L (55% of the predicted value), residual volume/predicted value 149%. Based on the patient’s examination results, shortness of breath caused by polymyositis or heart disease was excluded. The patient’s PaO_2_ was obviously improved after deep breathing. Considering that there was no obstructive ventilation function disturbance, the decrease in FVC may have been related to neuromuscular myopathy. Experts from the neurology department were invited for consultation.

Further examination after neurology consultation indicated no abnormality in tumor markers (AFP, CA242, SCCAg, PSA, CEA, CA199, CA125, CA724, CA153, NSE, TPS, Cyfra211, ProGRP), antibodies of the anti-Hu, anti-Yo and anti-Ri type, blood and urine immunofixation electrophoresis, serum protein electrophoresis, blood homocysteine, folic acid, vitamin B12 or thyroid function. No abnormal signals were found on lower limb MRI of the muscles of each group, and the muscle space was clear. Nervous system damage caused by paraneoplastic syndrome, autoimmune diseases and folic acid and vitamin B12 deficiency were excluded. Considering the obvious increase in blood lactate after exercise, further electromyography and muscle biopsy were proposed to determine whether nerve conduction and muscle mitochondrial lesions were present. However, due to the impact of the corona virus disease 2019 pandemic, the examinations could not be carried out and were scheduled for the future. The preliminary diagnosis after the neurology consultation was hypoxemia and possible mitochondrial myopathy. The patient was treated with vitamin B1 10 mg three times daily, vitamin B complex 1 tablet three times daily and methycobal 0.5 mg three times daily for nerve nourishment and with aerosol inhalation administered with budesonide suspension, 1 mg/time, twice daily. Entecavir was continued for hepatitis B treatment.

After the above treatments, the patient’s symptoms were not improved, and even showed occasional worsening. In May 2020, the patient entered a coma and had blood gas values of pO_2_ 20–30 mmHg ↓ and pCO_2_ 83 mmHg ↑. He underwent tracheotomy and ventilator-assisted ventilation at the local hospital. On July 2, blood gas review after the patient’s condition had stabilized showed pH 7.38, PaO_2_ 57.9 mmHg ↓, PaCO_2_ 48.1 mmHg ↑, and lactate 3.5 mmol/L ↑, indicating improvement. For a definitive diagnosis, the patient visited the Department of Neurology, Peking Union Medical College Hospital, for examination. The following results were obtained, electromyography: the upper and lower limb nerves and parasternal muscles showed normal conduction. Pathological report for the quadriceps femoris biopsy suggested myogenic changes, slight differences in the sizes of muscle fibers, mild atrophy in scattered muscle fibers, light cytochrome c oxidase staining in a few muscle fibers, and suspicious ragged red fibers on modified Gomori trichrome staining (The patient hadn’t muscle biopsy result prior to initial entecavir treatment). Mitochondrial myopathy was diagnosed on July 11. Considering that mitochondrial myopathy is associated with entecavir, entecavir discontinuation was recommended.

After entecavir had been discontinued for 1 month, the patient’s shortness of breath was improved. A review indicated HBV DNA 1E + 03 IU/ml and normal ALT. A blood gas review carried out 4 months after the discontinuation of entecavir (November 17) showed obviously improved results: PaO_2_ 77.9 mmHg ↓, PaCO_2_ 39.7 mmHg, lactate 2.4 mmol/L ↑. However, some parameters had worsened: ALT 333 u/L ↑, aspartate aminotransferase (AST) 153 u/L ↑ and HBV DNA 8 E + 07 IU/ml. Therefore, the patient visited a clinic at Beijing Youan Hospital on December 3, 2020, for treatment. Due to virological rebound and abnormal biochemistry, reactivation of hepatitis B was considered. The patient was recommended to take tenofovir alafenamide 1 tablet (25 mg) once a day for treatment. Meanwhile, his respiratory symptoms and mitochondrial myopathy were closely monitored. A follow-up visit in March 2021 found that the patient did not take the tenofovir alafenamide due to concerns about the risk of tenofovir alafenamide-induced respiratory failure and had only received diammonium glycyrrhizinate and polyene phosphatidylcholine for treatment. His liver function indexes had decreased (ALT 81.1 u/L ↑, AST 71.4 u/L ↑, HBV DNA 8 E + 07 IU/ml) but were still not recovered. At that time, entecavir treatment had been discontinued for 8 months. His dysosmia had improved slightly (pungent smells could be identified). Symptoms including shortness of breath, fatigue, poor appetite, etc., were improved. Blood lactate had decreased to 2.1 mmol/L↑. Compared previous measurements, the serum muscle zymogram had not decreased: CK-MB 42.6 u/L ↑, LDH 350 u/L ↑, HBDH 309 u/L↑, CK 222. The electrocardiogram was still normal.

The last follow-up was in August 2021. The patient’s shortness of breath and fatigue were alleviated, and he could tolerate activities of daily living. However, the tracheotomy needed to be kept open when the patient was lying flat at night. The patient’s liver function indexes had still not completely reduced to normal (ALT 33.2 u/L, AST 47.1 u/L ↑). HBV DNA was 8.97E + 07 IU/ml. The serum muscle zymogram were decreased (CK-MB 40.9 u/L ↑, LDH 287 u/L↑, HBDH 278 u/L↑, CK 154 u/L). Blood lactate was decreased to normal (1.3 mmol/L).

## Discussion and conclusions

Domestic and international guidelines for hepatitis B all recommend that CHB patients should receive long-term treatment with NAs to reduce recurrence caused by drug withdrawal. However, with the long-term use of NAs, reports of NAs-related mitochondrial myopathy have gradually increased, although most cases have been reported in patients treated with telbivudine, lamivudine or adefovir and mainly manifest as myasthenia of the limbs and myalgia [1, 5–9]. Cases with involvement of the respiratory muscles with respiratory failure as the prominent manifestation and accompanying dysosmia have not been reported.

As early as 2009, our team found that the use of telbivudine combined with interferon to treat CHB could cause peripheral neuritis accompanied by significantly increased AST [159.6 u/L (77.5, 299.4)] and ALT [72.4 u/L (60.6, 98)] with DNA < 500 copies/ml. Some cases showed abnormal CK and CK-MB or even degenerative changes in the toenails [10]. Therefore, interferon and telbivudine are no longer recommended for combination treatment. However, with the extension of NAs monotherapy, reports that NAs can lead to mitochondrial myopathy have gradually emerged. The main mechanism may be that NAs not only inhibit HBV DNA polymerase but also affect human mitochondrial DNA polymerase, resulting in insufficient synthesis of respiratory chain-related enzymes, energy metabolism disorder and, ultimately, mitochondrial myopathy [4]. Mitochondrial lesions can affect the tissues and organs of the whole body, and the abundant mitochondria in brain, nerve and muscle tissues make them vulnerable due to their high dependence on oxidative metabolism. Relevant previous studies have mostly reported isolated mitochondrial myopathy, i.e., mitochondrial myopathy involving only the skeletal muscle system. In the case reported in this study, in addition to the commonly observed involvement of the limb muscles of the skeletal muscle system, severe respiratory muscle involvement was also observed. In addition, the involvement of multiple systems, including the nervous system and digestive system, were also found. The patient was not diagnosed until 5 years after the onset of the disease, after visiting multiple hospitals for treatment, which is rare.

Although the patient’s blood gas test results indicated type II respiratory failure, hypoxemia and CO_2_ retention were significantly improved after deep breathing, indicating that there was no obstructive disturbance of ventilatory function. The patient had no history of smoking or heart and lung diseases, and ancillary examinations showed that thoracic muscle function and nerve conduction were normal. The restrictive disturbance in ventilatory function may have been caused by respiratory muscle dyskinesia. In addition to muscle involvement, this patient also had olfactory nerve involvement, which is rare. Pathological changes in the mitochondria, an important energy supply factory in the body, can affect multiple organs and systems. Therefore, for CHB patients who have undergone long-term treatment with NAs, the possibility of mitochondrial myopathy should be considered if there are clinical symptoms that are difficult to explain with routine examinations.

In previous reports of 3 cases of entecavir-associated myopathy, the onset time ranged from 10 days to 5 years, and the manifestations were only myalgia or myasthenia of the limbs. In these 3 cases, CK and/or LDH and HBDH increased to different degrees, and the symptoms of myopathy were gradually relieved and kinase levels were decreased after withdrawal of the drug [3, 11, 12]. Among the 3 cases, 2 developed rebound of the hepatitis B virus and increased transaminase after withdrawal of the drug. After treatment with lamivudine plus adefovir for 2 months [11] and lamivudine alone for 1 month [12], the liver function and HBV DNA of these 2 cases returned to normal, and no adverse reactions occurred during this treatment period. However, there were no follow-up reports regarding the subsequent long-term treatment of these cases or whether myopathy recurred. Our patient also experienced hepatitis reactivation after the withdrawal of entecavir, which was in line with the current indications of hepatitis B antiviral treatment. However, both NAs and interferon have treatment risks. The former may lead to the recurrence of respiratory failure, while the latter may cause additional adverse reactions during clinical application. The patient consulted a neurologist, and antiviral treatment was not recommended. However, the patient has been treated with hepatoprotective drugs for more than 8 months, and his transaminase level has never recovered. If the liver is repeatedly inflamed over a long period, liver failure and cirrhosis may occur. Therefore, it is still unknown how the patient should be treated in the future.

In summary, the long-term use of entecavir for CHB can cause mitochondrial myopathy. Although the incidence is extremely low, the disease is very serious and should receive adequate attention in clinical practice.

## Data Availability

All data generated or analyzed during this study are included in this published article.

## Abbreviations

NAs: Nucleos(t)ide analogue
CHB: Chronic hepatitis B
HBV: Hepatitis B virus
HBsAg: Hepatitis B surface antigen
HBeAg: Hepatitis B e antigen
CT: Computed tomography
MRI: Magnetic resonance imaging
PaO_2_: Arterial oxygen partial pressure
PaCO_2_: Arterial carbon dioxide partial pressure
CK-MB: Creatine kinase-myocardial band
LDH: Lactate dehydrogenase
HBDH: Hydroxybutyrate dehydrogenase
CK: Creatine kinase
FEV1: Forced expiratory volume in 1 s
FVC: Forced vital capacity
SO_2_: Oxygen saturation
AST: Aspartate aminotransferase

## References

[1] Xu H, Wang Z, Zheng L (2014). Lamivudine/telbivudine-associated neuromyopathy: neurogenic damage, mitochondrial dysfunction and mitochondrial DNA depletion. J Clin Pathol.

[2] Wang M, Da Y, Cai H (2012). Telbivudine myopathy in a patient with chronic hepatitis B. Int J Clin Pharm.

[3] Cai-Hong QU, Zhu JM, Zhang YF (2013). Drug induced myopathy associated with entecavir in one patient with chronic hepatitis B. Chin J New Drugs Clin Rem.

[4] Kayaaslan B, Guner R (2017). Adverse effects of oral antiviral therapy in chronic hepatitis B. World J Hepatol.

[5] Adani GL, Baccarani U, Risaliti A (2005). Rhabdomyolysis due to lamivudine administration in a liver transplant recipient. Am J Transplant.

[6] Yahagi K, Ueno Y, Mano Y (2002). Rhabdomyolytic syndrome during the lamivudine therapy for acute exacerbation of chronic type B hepatitis. Liver Transpl.

[7] Ambang T, Tan JS, Ong S (2016). Clinicopathological features of telbivudine-associated myopathy. PLoS ONE.

[8] Zou S, Cheng Z, Song S (2019). Telbivudine-induced myopathy incidentally detected by FDG PET/CT imaging in a patient with history of hepatocellular carcinoma. Clin Nucl Med.

[9] Fujii T, Takase KI, Honda H (2019). Toxic myopathy with multiple deletions in mitochondrial DNA associated with long-term use of oral anti-viral drugs for hepatitis B: a case study. Neuropathology.

[10] Chen X, Wu Y, Ma L (2009). Clinical analysis of telbivudine or combined with interferon in the treatment of chronic hepatitis B complicated with peripheral neuritis. Chin J Infect Dis..

[11] Yuan K, Guochun W, Huang Z (2014). Entecavir-associated myopathy: a case report and literature review. Muscle Nerve.

[12] Chen J (2014). Entecavir causes severe myalgia: a case report. Chin Hepatol.

